# Praziquantel treatment after *Schistosoma japonicum* infection maintains hepatic insulin sensitivity and improves glucose metabolism in mice

**DOI:** 10.1186/s13071-017-2400-5

**Published:** 2017-10-02

**Authors:** Xiaofeng Luo, Yuxiao Zhu, Ran Liu, Jingwei Song, Fan Zhang, Wenyue Zhang, Zhipeng Xu, Min Hou, Bingya Yang, Lin Chen, Minjun Ji

**Affiliations:** 1 Department of Pathogen Biology, Nanjing Medical University, Nanjing, Jiangsu 211166 China; 2 Jiangsu Province Key Laboratory of Modern Pathogen Biology, Nanjing, Jiangsu 211166 China

**Keywords:** *Schistosoma japonicum*, Glucose metabolism, Insulin resistance, Anti-inflammatory cytokines

## Abstract

**Background:**

Epidemiological studies in China have revealed that *Schistosoma japonicum* infection is inversely correlated with metabolic syndrome, even after repeated chemotherapy with praziquantel (PZQ). We investigated the effect of chronic *S. japonicum* infection, PZQ chemotherapy, and soluble egg antigen (SEA) treatment on whole-body metabolic homeostasis and hepatic insulin sensitivity in mouse models.

**Results:**

Infection with *S. japonicum* was found to increase whole-body and hepatic insulin sensitivity in mice. PZQ chemotherapy significantly improved the physiological status of infected mice, maintaining Th2 immune-deviation and enhancing hepatic insulin sensitivity. Multiple linear regression analysis revealed positive correlations between anti-inflammatory cytokine expression and insulin signalling-related genes in the liver, as demonstrated by an *in vitro* stimulated hepatic cell line with IL-13 and IL-22. SEA treatment also improved the glucose tolerance and insulin sensitivity in *Lepr* ^*db/db*^ mice.

**Conclusions:**

This study indicated that chronic *S. japonicum* infection with PZQ chemotherapy and SEA treatment can regulate metabolic homeostasis and protect against metabolic syndrome by promoting Th2 and regulatory responses in the liver.

**Electronic supplementary material:**

The online version of this article (10.1186/s13071-017-2400-5) contains supplementary material, which is available to authorized users.

## Background

In recent years, obesity, type 2 diabetes mellitus (T2DM), dyslipidaemia and their associated metabolic syndrome (MetS) have been become severe public health issues. Excess consumption of high-energy foods and decreased physical activity are two main factors contributing to metabolic syndrome [1, 2]. Additionally, studies have found that the decreased incidence of parasitic diseases is associated with a significant increase in the prevalence of chronic inflammatory disorders, including asthma, inflammatory bowel disease, some autoimmune diseases (e.g. type 1 diabetes and multiple sclerosis) and metabolic dysfunction (e.g. type 2 diabetes and atherosclerosis) [3]. Correale et al. [4] compared helminth-infected and uninfected patients with multiple sclerosis and found a significantly lower number of exacerbations, minimal variation in disability scores and fewer magnetic resonance imaging (MRI) changes in infected individuals over a 4.6-year follow-up period. Furthermore, when some infected patients received anthelmintic treatment for their intestinal symptoms, the number of clinical relapses and MRI changes increased, whereas the number of the regulatory immune responses decreased [5].

In rural areas of Indonesia, blood lipid levels in individuals with intestinal parasitic infections were found to be significantly lower than in those without infection [6]. A report from the Chennai Urban Rural Epidemiology Study (CURES) in India showed that patients with type 2 diabetes had a lower prevalence of lymphatic filariasis than did non-diabetic and pre-diabetic subjects, suggesting that the decreased filarial infection potentially had an unexpected adverse impact on diabetes prevalence. Moreover, filarial-positive diabetic subjects had lower levels of pro-inflammatory markers (TNF-α, IL-6 and GM-CSF) than did those who were filarial-negative [7]. These findings suggest that helminth infections play critical roles in protecting against some autoimmune and metabolic disorders.

Over the past 60 years, the Chinese government has adopted a series of policies and measures to control schistosomiasis japonica [8]. Most endemic areas have reached the criteria for transmission-control and transmission-block. We found that patients with a previous schistosome infection (PSI) living in the suburb of the Shanghai municipality or the cities of Suzhou and Wuxi in Jiangsu Province, China, which used to be endemic areas for schistosomiasis japonica, had a lower incidence of metabolic syndrome and complications, compared to those without PSI, based on reports from 2013 [9] and 2015 [10]. These findings suggests that individuals with PSI, even after repeated treatment with praziquantel (PZQ), can maintain a prolonged period of relatively low metabolic symptoms. Schistosome-associated liver morbidity contributes to low energy metabolism in the host, and helminth-driven polarized immune and regulatory processes can account for long-term metabolic adaptation in endemic populations [11]. During schistosome infection, there is a shift from a Th1- to Th2-dominant immune response with a large number of eggs appearing and a gradual development of immune regulation in the late stages of the disease. This diminishes the strength of the inflammatory response [12–14]. Metabolic syndrome, including T2DM and obesity, is considered an inflammatory disease, and its progression involves inflammatory factors such as TNF-α, IL-6 [15, 16] and C-reactive protein. Thus, schistosome infections and metabolic syndrome are almost mutually exclusive.

To investigate the impact of schistosome infections and PZQ chemotherapy on host energy metabolism, we established a mouse model of *Schistosoma japonicum* chronic infection and PZQ chemotherapy, analysed the cytokine profiles at the different infection and treatment stages, and assessed the glucose metabolism in parallel. Furthermore, we treated C57BL/6 and *Lepr* ^*db/db*^ mice with soluble egg antigens (SEA) to observe the potential therapeutic effects on hyperglycaemia.

## Methods

### Animals, parasites and antigen preparation

Male C57BL/6 mice and the diabetes db mutation of the leptin receptor (*Lepr* ^*db/db*^) mice (aged 6 weeks) were used in all experiments. Mice were obtained from the Model Animal Research Centre of Nanjing University and kept in a specific pathogen-free environment at the Animal Care Facility of Nanjing Medical University.

Snails of the Chinese strain of *S. japonicum*-infected *Oncomelania hupensis*, were bought from the Jiangsu Institute of Parasitic Diseases (Wuxi, China). Cercariae were collected from infected snails.

SEA was prepared carefully to prevent endotoxin contamination [17]. The concentration of SEA was assayed using the Bicinchoninic Acid Protein Assay Kit (Pierce, Rockford, IL, USA). The endotoxin concentration of the SEA was < 0.03 EU⁄ml as measured by a timed gel endotoxin detection kit (Sigma, St. Louis, MO, USA).

### Experimental mice models

#### Infection-chemotherapy model

Ninety mice were included in this experiment and randomly divided into three groups. Sixty mice were infected with 10 ± 2 *S. japonicum* cercariae. At the sixth week post-infection, PZQ was dissolved in sodium carboxymethyl cellulose (CMC) and administered to 30 mice by gavage at 150 mg/kg/day for two consecutive days to comprise the PZQ chemotherapy group (C57BL/6-inf-PZQ group). Another 30 infected mice were administered an equal volume of CMC and formed the CMC vehicle group (the chronic infection group or C57BL/6-inf-CMC group). Thirty untreated mice were designated the control group (C57BL/6-con group). Metabolic studies started from the time of PZQ or CMC treatment. The fasting blood glucose, glucose-tolerance test (GTT) and insulin-tolerance test (ITT) were conducted after mice were fasted for 6–12 h at 3, 6, 9 and 12 weeks after treatment. Additionally, six mice from each group were sacrificed at 0, 3, 6, 9 and 12 weeks after treatment, respectively. Serum and liver tissue were collected to measure the levels of lipids, insulin, inflammatory cytokine gene transcription, and insulin sensitivity pathway targets in the blood, as well as phosphorylated Akt (p-Akt) expression in the liver.

#### SEA treatment model

We conducted similar metabolic experiments in the C57BL/6 mice and the diabetes db mutation of the leptin receptor (*Lepr* ^*db/db*^) mice (*n* = 5 per treatment) and subjected them to repetitive intraperitoneal injections with 0.9% NaCl (vehicle) or SEA dissolved in 0.9% NaCl (25 μg, twice a week for 6 weeks). Metabolic studies including GTT and ITT were conducted at 4 and 6 weeks after SEA treatment. Mice were sacrificed at 6 weeks after SEA treatment for serum and liver tissue collection to test the blood fasting insulin concentration and gene transcription in the liver.

### Body weight, food intake and water intake

Basic physiological parameters including body weight, food intake and water intake were monitored weekly (three times per week) from the first week after PZQ and SEA treatment.

### Blood biochemistry

Serum alanine aminotransferase (ALT), aspartate aminotransferase (AST), total cholesterol (TC), triglycerides (TG), high-density lipoprotein cholesterol (HDL-C), and low-density lipoprotein cholesterol (LDL-C) were measured using an automatic biochemical analyser.

### Glucose and insulin-tolerance tests

GTT was performed in mice after a 12 ~ 16-h overnight fast. After determining the fasted blood glucose levels, animals received an intraperitoneal (i.p*.*) injection of 2 g glucose per kg body weight. Blood glucose levels were measured before and after injection at 15, 30, 60 and 120 min using a glucose monitoring system. ITT was conducted similarly (fasting 6–8 h) by intraperitoneally injecting 0.75 U per kg body weight of insulin. *Lepr* ^*db/db*^ animals received 5 U per kg of insulin.

### Serum insulin levels

Serum insulin levels were measured by enzyme-linked immunosorbent assay (ELISA) as per the instruction manual (Mouse/Rat Insulin ELISA Kit, EMD Millipore Corporation, Billerica, MA, USA). Blood glucose levels were measured weekly with blood from the caudal vein using an automatic glucose monitor, and the homeostasis model of assessment-insulin resistance (HOMA-IR) was calculated.

### Immunoblotting experiments

*In vivo* insulin signalling was determined by injecting 0.75 U/kg insulin per C57BL/6 mouse or 5 U/kg insulin per *Lepr* ^*db/db*^ mouse in the portal vein. We collected liver samples before and 15 min after insulin injection and rapidly froze tissues in liquid nitrogen for storage.

We performed western blot analyses using different antibodies to detect the following proteins: p-Akt (Cell Signaling, Danvers, MA, USA #12694, 1:1000), t-Akt (Cell Signaling, Danvers, MA, USA #2920, 1:2000) and β-Actin (Abmart, Shanghai, China #P30002, 1:2000).

### *In vitro* experiments

TNF-α, IL-13 and IL-22 were used to stimulate the mouse hepatic cell line (FL83B), which was obtained from the Liver Transplantation Center of Jiangsu Province Hospital. The corresponding concentrations and times were 20 ng/ml and 24 h for TNF-α, 50 ng/ml and 24 h for IL-13, and 40 ng/ml and 12 h for IL-22. The expression of metabolism related indicators such as insulin receptor substrate 1(IRS-1), insulin receptor substrate 2 (IRS-2), insulin receptor (INSR), glucose-6-phosphatase (G6PC) and glucose transporter 4 (GLUT4) was detected by q-PCR. The mean and SEM were determined from 3 biological replicates for each representative experiment. Experiments were repeated at least three times.

### Real-time quantitative PCR analysis

For gene expression analyses, we determined the relative expression levels of designated inflammatory or anti-inflammatory cytokines (TNF-α, IL-6, IL-1β, TGF-β1, IL-10, IL-13, IL-22 and IL-33) and insulin sensitivity pathway related genes (IRS-1, IRS-2, INSR, G6PC and GLUT4) using SYBR green-based real-time quantitative PCR (q-PCR) reactions. The relative expression of the mRNA was calculated using a comparative method (2^-△△^ct) according to the ABI Relative Quantification Method. The primers used are shown in Table 1.

**Table 1:** Real-time quantitative PCR primer sequences

Gene	Direction	Sequence (5′-3′)
GAPDH	Forward	AACTTTGGCATTGTGGAAGG
	Reverse	GGATGCAGGGATGATGTTCT
IRS-1	Forward	GGTCACGAAGAGCTGGTGCACT
	Reverse	GAGGCGCATCATGGAACACGG
IRS-2	Forward	GCTCCCTGTTCCTGCAGCGG
	Reverse	CAAAGGTGCCAGCCCCTGGG
INSR	Forward	GATGTGCACCCCATGTCTG
	Reverse	CTGAATAGCTGAGACCACAG
G6PC	Forward	TTACCAAGACTCCCAGGACTG
	Reverse	GAGCTGTTGCTGTAGTAGTCG
GLUT4	Forward	TGTATGTGGGAGAAATCGC
	Reverse	GACAGAAGGGCAGCAGAAT
TNF-α	Forward	CATCTTCTCAAAATTCGAGTGACAA
	Reverse	TGGGAGTAGACAAGGTACAACCC
IL-6	Forward	GAGGATACCACTCCCAACAGACC
	Reverse	AAGTGCATCATCGTTGTTCATACA
IL-1β	Forward	GCACACCCACCCTGCA
	Reverse	ACCGCTTTTCCATCTTCTTCTT
TGF-β1	Forward	ATGCTAAAGAGGTCAAAAGC
	Reverse	CCAAGGTAACGCCAGGAATT
IL-10	Forward	ACTTTAAGGGTTACTTGGGTTGC
	Reverse	ATTTTACAAGGGGAGAAATCG
IL-13	Forward	GCCAGCCCACAGTTCTAC
	Reverse	GAGATGTTGGTCAGGGAAT
IL-22	Forward	GTCAACCGCACCTTTATGCT
	Reverse	CATGTAGGGCTGGAACCTGT
IL-33	Forward	GATGGGAAGAAGGTGATGGTG
	Reverse	TTGTGAAGGACGAAGAAGGC

### Statistical analyses

All statistical analyses were conducted using SPSS software (version 19.0; SPSS Inc., Chicago). Comparisons among the three groups were performed by one-way ANOVA, and the LSD test was used for comparisons between two groups. Two-way ANOVA was used to determine statistical significance for GTT and ITT. The hepatic transcription level dependence of the metabolic indicators, IRS-1, IRS-2, INSR, G6PC, and GLUT4 on TNF-α, IL-6, IL-1β, TGF-β1, IL-10, IL-13, IL-22, and IL-33 in the *S. japonicum* chronic infection and PZQ chemotherapy groups was analysed by multiple linear regression analysis. Values are presented as the mean ± standard error (SE); *P* < 0.05 was considered significant.

## Results

### PZQ treatment significantly improved physiological status of infected mice with *S. japonicum*

**Fig. 1:**
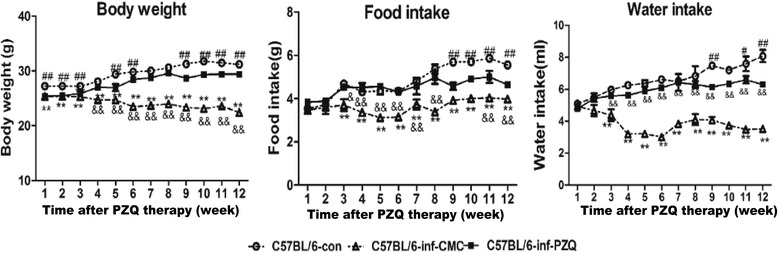
Dynamic curves for body weight, food intake and water intake after PZQ or CMC treatment in Infection-Chemotherapy model mice. Statistical comparisons are indicated as follows: *C57BL/6-inf-CMC group *vs* C57BL/6-con group; # C57BL/6-inf-PZQ group *vs* C57BL/6-con group; & C57BL/6-inf-PZQ group *vs* C57BL/6-inf-CMC group. # *P* < 0.05; **, ## and && *P* < 0.01

To study the effect of chronic *S. japonicum* infection and PZQ treatment on whole-body physiological homeostasis, we detected body weight, food intake and water intake weekly starting at 6 weeks post-infection. As shown in Fig. 1, compared with normal mice, body weight, food intake and water intake in the chronically infected mice were markedly decreased (*F*_(2,6)_ = 1291, *P* < 0.0001; *F*_(2,6)_ = 813.1, *P* < 0.0001; *F*_(2,6)_ = 266.8, *P* < 0.0001, respectively) (Fig. 1). However, PZQ treatment at 6 weeks post-infection significantly improved the physiological status of the infected mice, as represented by an increase in body weight, food intake and water intake for mice in the C57BL/6-inf-PZQ group compared to those in the C57BL/6-inf-CMC group. Mice receiving PZQ treatment ate and drank less than normal mice did after 9 weeks post-treatment. In addition, PZQ treatment significantly alleviated hepatic pathology. The granulomatous area containing a single egg was reduced after PZQ treatment (Additional file 1: Figure S1).

### PZQ treatment improved whole-body glucose tolerance and insulin sensitivity

**Fig. 2:**
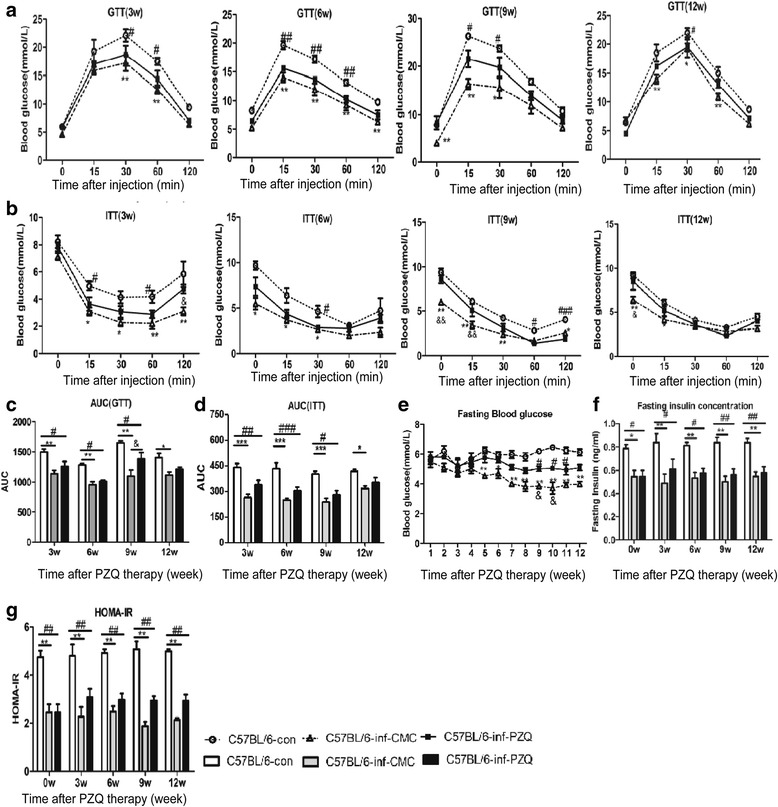
Dynamic changes in blood glucose levels after PZQ or CMC treatment in Infection-Chemotherapy model mice. **a** Glucose tolerance test at 3, 6, 9 and 12 weeks after PZQ or CMC treatment. **b** Insulin tolerance test at 3, 6, 9 and 12 weeks after PZQ or CMC treatment. **c** Area under the curve of GTT. **d** Area under the curve of ITT. **e** Fasting blood glucose. **f** Serum insulin concentration. **g** Homeostasis model of assessment-insulin resistance (HOMA-IR). Statistical comparisons are indicated as follows: * C57BL/6-inf-CMC group *vs* C57BL/6-con group; # C57BL/6-inf-PZQ group *vs* C57BL/6-con group; & C57BL/6-inf-PZQ group *vs* C57BL/6-inf-CMC group. *, # and & *P* < 0.05; **, ## and && *P* < 0.01

The glucose tolerance test, insulin tolerance test, area under the curve (AUC), fasting insulin concentrations and HOMA-IR were used to assess glucose tolerance and insulin sensitivity in the Infection-Chemotherapy model mice. When subjected to the glucose tolerance test, PZQ and CMC (vehicle) treated mice at 3, 6, 9 and 12 weeks after PZQ or CMC injection showed significantly higher glucose tolerance compared with the control group (Fig. 2a). When subjected to the insulin tolerance test, mice who received PZQ and CMC treatment also had improved insulin sensitivity compared with the control group (Fig. 2b). Figure 2c, d show that the AUC of GTT and ITT in mice with *S. japonicum* chronic infection were significantly less than the AUC in control mice at 3, 6, 9 and 12 weeks after CMC treatment. A similar pattern was observed in mice with PZQ chemotherapy at 3, 6 and 9 weeks post-treatment, probably due to egg antigens still being present in the liver post PZQ chemotherapy at 6 weeks post-infection (*F*_(2,12)_ = 45.52, *P* < 0.0001; *F*_(2,12)_ = 56.28, *P* < 0.0001) (Fig. 2c, d). However, there was no significant difference in glucose metabolism between the PZQ treatment and chronic infection groups until 12 weeks post-treatment. As demonstrated by the fasting serum insulin concentrations and HOMA-IR reductions in the chronic infection and PZQ chemotherapy groups, schistosome infection and PZQ treatment could improve and maintain insulin sensitivity in the body (Fig. 2f, g). In addition, the analyses of ALT, AST, TC, TG, LDL-C and HDL-C suggested that *S. japonicum* infection and PZQ treatment can improve liver function and maintain a relatively low lipid metabolism in mice (Additional file 2: Figure S2).

### PZQ treatment improved hepatic insulin sensitivity

**Fig. 3:**
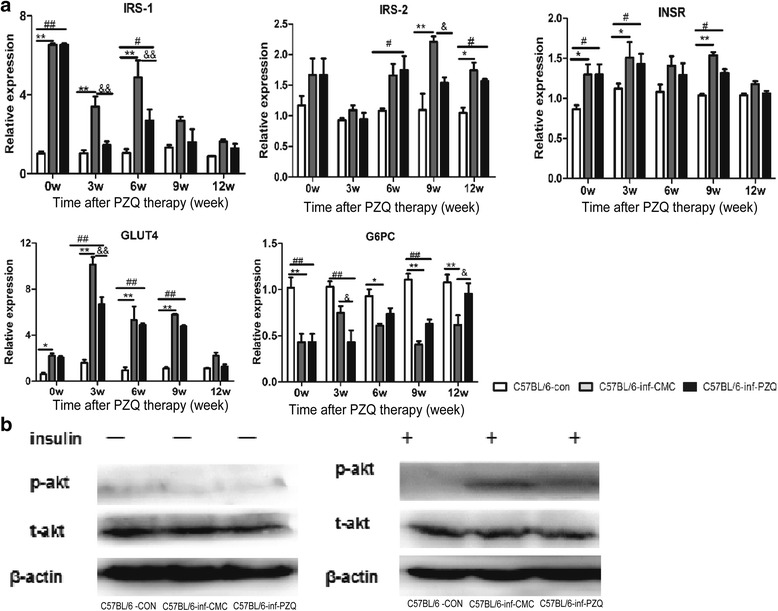
Dynamic gene expression of insulin sensitivity pathway targets in the liver after PZQ or CMC treatment in Infection-Chemotherapy model mice. **a** mRNA transcription of glucose and insulin sensitivity pathway related genes after PZQ or CMC treatment, detected by real-time quantitative PCR. **b** Insulin stimulated Akt phosphorylation in livers from vehicle and PZQ treated mice examined by western blot (p-Akt: phospho-Akt; t-Akt: total Akt). Statistical comparisons are indicated as follows: * C57BL/6-inf-CMC group *vs* C57BL/6-con group; # C57BL/6-inf-PZQ group *vs* C57BL/6-con group; & C57BL/6-inf-PZQ group *vs* C57BL/6-inf-CMC group. *, # and & *P* < 0.05; **, ## and && *P* < 0.01

To ascertain the interaction between hepatic insulin sensitivity and chronic *S. japonicum* infection or PZQ treatment, we detected gene transcription in designated targets involved in the insulin sensitivity pathway in the liver. Consistent with the increased whole-body insulin sensitivity, schistosome infection significantly upregulated the expression of IRS-1, IRS-2, INSR, and GLUT4 and downregulated G6PC expression, particularly at 0–9 weeks after CMC treatment. Infected mice receiving PZQ treatment maintained similar gene expression trends in the liver as the mice with chronic infection (*F*_(2,9)_ = 61.5, *P* < 0.0001; *F*_(2,9)_ = 19.29, *P* = 0.0006; *F*_(2,9)_ = 7.294, *P* = 0.0131; *F*_(2,9)_ = 208.2, *P* < 0.0001; *F*_(2,9)_ = 42.40, *P* < 0.0001) (Fig. 3a).

To further observe the effect of the *S. japonicum* chronic infection and PZQ treatment on hepatic specific insulin sensitivity, mice were subjected to an acute hepatic portal vein insulin injection to enhance insulin signalling at 9 weeks after PZQ or CMC treatment, and insulin-stimulated Ser473 Akt phosphorylation in the liver was examined by western blot analyses. We obtained similar results showing that Akt phosphorylation protein levels were both elevated in the CMC and PZQ treated mice (Fig. 3b). These results suggest that chronic *S. japonicum* infection promotes hepatic insulin signalling, as does PZQ treatment.

### PZQ treatment increased IL-13 and IL-22 expression in the liver

**Fig. 4:**
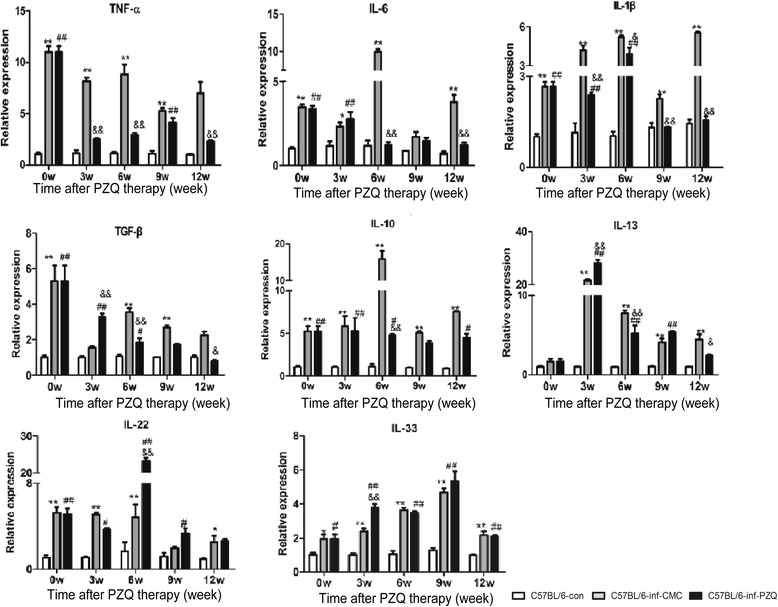
Dynamic mRNA transcription of inflammatory and anti-inflammatory cytokines in the liver after PZQ or CMC treatment in Infection-Chemotherapy model mice. Statistical comparisons are indicated as follows: * C57BL/6-inf-CMC group *vs* C57BL/6-con group; # C57BL/6-inf-PZQ group *vs* C57BL/6-con group; & C57BL/6-inf-PZQ group *vs* C57BL/6-inf-CMC group. *, # and & *P* < 0.05; **, ## and && *P* < 0.01

Because inflammation plays a crucial role in schistosome infection and host metabolism, we detected the expression of pro-inflammatory and anti-inflammatory cytokines in Infection-Chemotherapy model mice. Compared with control mice, the expression of pro-inflammatory cytokines such as TNF-α, IL-6, and IL-1β was significantly increased in both *S. japonicum* chronically infected and PZQ-treated mice. More importantly, mice in these two groups showed significantly high expression of many anti-inflammatory cytokines such as TGF-β, IL-10, IL-13, IL-22 and IL-33, and hepatic expression of IL-13 and IL-22 was higher in PZQ-treated mice than in chronically infected mice (*F*_(2,9)_ = 327.8, *P* < 0.0001; *F*_(2,9)_ = 154.5, *P* < 0.0001) (Fig. 4). These results show that PZQ treatment can alleviate inflammation by down-modulating the expression of pro-inflammatory cytokines and upregulating the expression of anti-inflammatory cytokines.

To explore the roles of these inflammatory and anti-inflammatory cytokines in hepatic insulin signalling during *S. japonicum* infection and PZQ chemotherapy, we implemented multiple linear regression analysis between the cytokines and target genes involved in the insulin and glucose pathways. In the *S. japonicum* chronically infected mice, IL-22 expression was positively correlated with INSR and GLUT4, and IL-33 expression was positively correlated with IRS-2 and GLUT4. The expression of both IL-22 and IL-33 negatively correlated with G6PC. In the PZQ-treated mice, the expression of IL-13 and IL-22 was positively correlated with IRS1 and GLUT4. These data suggest that anti-inflammatory cytokines contribute to increasing insulin sensitivity and accelerating glucose metabolism in the liver (Additional file 3: Tables S1–S3).

### SEA improved whole-body metabolic homeostasis in *Lepr*^*db/db*^ mice

**Fig. 5:**
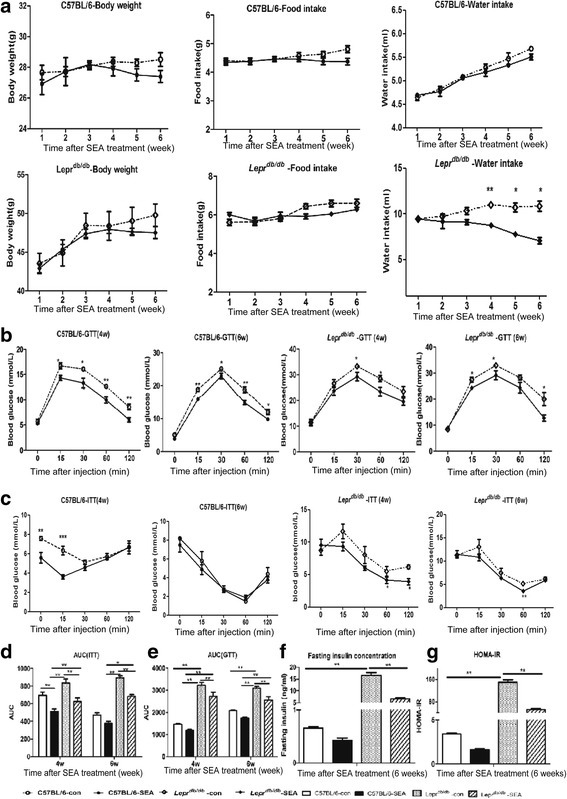
Body weight, food intake, water intake, GTT, ITT, fasting insulin concentrations and HOMA-IR in SEA-treated model mice. **a** Body weight, food intake and water intake of C57BL/6, *Lepr* ^*db/db*^ mice and SEA-treated mice. **b** Glucose tolerance test at 4 and 6 weeks after SEA treatment. **c** Insulin tolerance test at 4 and 6 weeks after SEA treatment. **d** Area under the curve of GTT. **e** Area under the curve of ITT. **f** Serum insulin concentration. **g** Homeostasis model of assessment-insulin resistance (HOMA-IR). *P < 0.05, **P < 0.01

To study the impact of parasite-derived molecules, particularly from schistosome eggs, on whole-body metabolism homeostasis, we injected C57BL/6 and *Lepr* ^*db/db*^ mice with SEA to test the metabolic indicators. Compared with C57BL/6 control mice (C57BL/6-con), body weight, food intake, and water intake in SEA-treated C57BL/6 mice (C57BL/6-SEA) decreased slightly, but not significantly. However, water intake in *Lepr* ^*db/db*^ mice with SEA treatment (*Lepr* ^*db/db*^-SEA) decreased significantly compared with *Lepr* ^*db/db*^ mice without treatment (*Lepr* ^*db/db*^-con) (*F*_(1,5)_ = 70.26, *P* < 0.0001) (Fig. 5a). These results suggest that SEA treatment can relieve polydipsia caused by metabolic disorders.

To assess insulin sensitivity in the SEA treatment model, we performed a glucose tolerance test, insulin tolerance test, area under the curve analysis, fasting insulin concentration test and HOMA-IR. When subjected to GTT or ITT in C57BL/6 or *Lepr* ^*db/db*^ mice, SEA-treated mice showed significantly higher glucose tolerance and improved insulin sensitivity compared with the control mice (*F*_(1,8)_ = 16.99, *P =* 0.0033; *F*_(1,8)_ = 39.06, *P =* 0.0002; *F*_(1,8)_ = 10.27, *P =* 0.0125; *F*_(1,8)_ = 3.456, *P =* 0.1001; *F*_(1,8)_ = 30.05, *P =* 0.0006; *F*_(1,8)_ = 5.243, *P =* 0.0513; *F*_(1,8)_ = 4.404, *P =* 0.0691; *F*_(1,8)_ = 16.92, *P =* 0.0034) (Fig. 5b, c). The AUCs of GTT and ITT in the SEA treatment groups were less than those of their control groups (Fig. 5d, e). Combined with the reductions in fasting serum insulin concentrations and HOMA-IR in SEA-treated mice, particularly in SEA-treated *Lepr* ^*db/db*^ mice (Fig. 5f, g), these results suggest that parasite egg-derived molecules improve whole-body glucose tolerance and insulin resistance and coordinate metabolic homeostasis.

### SEA promoted a type 2 immune response and improved hepatic insulin sensitivity in *Lepr*^*db/db*^ mice

**Fig. 6:**
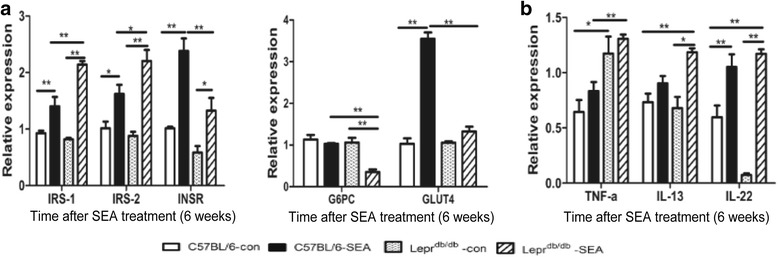
Gene expression of cytokines and targets in the insulin sensitivity pathway in the liver of SEA-treated mice. **a** Gene transcription of insulin sensitivity pathway related genes. **b** Gene transcription of some cytokines in SEA Treatment Model. *P < 0.05, **P < 0.01

To investigate the potential effect of SEA exposure on metabolic homeostasis, we detected the cytokine and target gene transcription in the glucose and insulin pathways in the liver. Compared to their respective controls, gene transcription of IRS-1, IRS-2, INSR and GLUT4 was significantly increased and G6PC expression was significantly decreased in livers from the SEA-treated C57BL/6 or *Lepr* ^*db/db*^ mice (*F*_(3,12)_ = 42.11, *P* < 0.0001; *F*_(3,12)_ = 18.01, *P* < 0.0001; *F*_(3,12)_ = 20.50, *P* < 0.0001; *F*_(3,12)_ = 18.36, *P* < 0.0001; *F*_(3,12)_ = 106.8, *P* < 0.0001, respectively) (Fig. 6a). Additionally, TNF-α expression was similar in the SEA-treated and untreated mice; however, the expression of anti-inflammatory cytokines, IL-13 and IL-22, was significantly higher with SEA treatment, primarily in the SEA-treated *Lepr* ^*db/db*^ mice (*F*_(3,8)_ = 9.310, *P* = 0.0055; *F*_(3,8)_ = 37.21, *P* < 0.0001, respectively) (Fig. 6b). Our data indicate that SEA promotes a type 2 immune response characterized by elevated anti-inflammatory IL-13 and IL-22 expression.

### IL-13 and IL-22 increased insulin sensitivity in the hepatic cell line

**Fig. 7:**
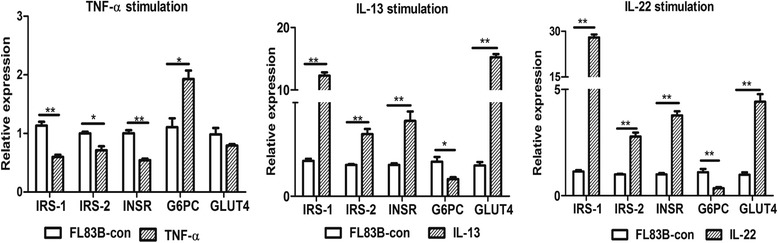
Gene expression in insulin sensitivity pathway targets in TNF-α, IL-13 and IL-22-stimulated hepatic cell lines. *P < 0.05, **P < 0.01

We used the cytokines TNF-α, IL-13, and IL-22 to stimulate the murine hepatic cell line (FL83B) and detected the expression of insulin signalling related genes. The IL-13 and IL-22 anti-inflammatory cytokines induced the expression of IRS-1, IRS-2, INSR, and GLUT4 and inhibited G6PC expression, and TNF-α did the opposite (Fig 7).

## Discussion

In recent years, many developing and underdeveloped countries have devoted efforts to deworming as a preventative measure for worm-associated morbidity. However, researchers are realizing that worm infections and certain immune and metabolic disorders mutually restrict each other; therefore, they are examining the therapeutic potential of worms and their secretory products to treat inflammatory diseases. Infections with certain helminths such as *Schistosoma mansoni* and *Necator americanus*, and protozoan parasites such as *Leishmania* and *Toxoplasma* [18], and their excretory/secretory products can manipulate host immune responses toward a Th2 immune phenotype. Emerging data suggests that helminths may help protect against MetS as well as autoimmune and allergic diseases [19, 20]. In studies on obese mice [11, 21], chronic infection with *S. mansoni* and treatment with SEA or immunomodulatory glycan LNFPIII, which is found in SEA, alleviated both hepatosteatosis and insulin resistance [11]. In the present study, chronic *S. japonicum* infection decreased glucose metabolism and upregulated insulin signalling in mice. Although food intake and spontaneous locomotor activity were not affected in mice with chronic *S. mansoni* infection, the relatively high pathogenicity of *S. japonicum* did lead to decreased food intake in mice who were in the late stages of the disease. In a mouse model of chronic *S. japonicum* infection, we found that at 6 weeks after egg-laying (24 days post-infection), the liver is suitable for studying the immunological correlation between the *S. japonicum* infection and host metabolism. In schistosomiasis japonica-endemic areas, infected individuals are often asymptomatic or mildly symptomatic; few hosts have overt clinical pathology with high worm burdens. Thus, based on epidemiological and laboratory studies, infection with *S. japonicum* is negatively correlated with metabolic disease despite the direct influence of *S. japonicum* on the host’s liver.

To avoid excess *S. japonicum* egg damage to the host’s liver, we treated *S. japonicum*-infected mice with PZQ at 6 weeks post-infection. Our study first investigated the effect on the host’s glucose metabolism after PZQ chemotherapy by dynamic continuous observation. PZQ chemotherapy improved the host’s physical condition in addition to maintaining other characteristics of the chronic *S. japonicum* infection, including a low glucose metabolism level, enhanced whole-body and hepatic insulin sensitivity and Th2-dominant immune responses. This result suggests that the existing schistosome eggs primed a Th2-dominant immune response and regulated the host’s metabolism and that this immune-deviation can be maintained for long periods even though PZQ kills the schistosome worms. This helps lain why individuals with a history of schistosomiasis and repeated PZQ treatments have a lower incidence of metabolic syndrome than do uninfected subjects in schistosomiasis japonica-endemic areas.

We examined *S. japonicum* eggs to determine if they secreted molecules that might have therapeutic potential against metabolic disease. Thus, we tested SEA to treat spontaneous diabetic mice (*Lepr* ^*db/db*^ mice). In our study, SEA injection increased whole-body and hepatic insulin sensitivity and modulated metabolic homeostasis and upregulated the expression of IL-13 and IL-22, particularly in the SEA-treated *Lepr* ^*db/db*^ mice. Our result was in accordance with that of the study on *S. mansoni* and high fat diet (HFD)-obese mice [11, 21]. Helminth-derived molecules can protect against metabolic disorders by promoting Th2 response, eosinophilia, and M2 polarization in white adipose tissue (WAT). A LewisX containing immunomodulatory glycan, LNFPIII from *S. mansoni*, was reported to improve glucose tolerance and insulin sensitivity in diet-induced obese mice through increased IL-10 production by LNFPIII-activated macrophages and dendritic cells [11]. This reduced WAT inflammation, sensitized the adipocyte insulin response, and increased the expression of nuclear receptor Fxr-α in the liver to suppress lipogenesis through the Erk-Ap1-Fxr-α axis [11]. We believe that other products derived from parasites might be worth exploring as potential therapeutic targets against metabolic diseases.

During long-term co-evolution, immunological interplay is a key factor in the partnership between helminths and their mammalian hosts. With schistosome infections, eggs drive the Th2-dominant immune response as well as other regulatory processes in the host, and these effects promote metabolic homeostasis. For example, IL-13 is a signature cytokine in the Th2 pathway, which is essential for granuloma formation, IgE production, basophilia, alternatively activated macrophage differentiation, and protection against fatal infection [22]. IL-6, IL-13 and IL-10 were found to be significantly elevated in *S. mansoni*-infected individuals in the Sudan [23]. Moreover, functional polymorphisms in IL-13 was found to be protective against high *S. mansoni* infection intensity in the Brazilian population [24]. IL-22 is a member of the IL-10 family of cytokines, which exerts an anti-inflammatory role in hepatitis and inflammatory bowel disease and plays an important role in host defence against infectious diseases, especially those caused by extracellular pathogens [25–28]. Some studies [29, 30] have reported that schistosome infection and egg stimulation can induce the production of IL-22 transcripts in schistosome-infected mice, and that schistosome eggs selectively stimulate IL-22 production in blood leukocyte cultures from individuals chronically infected with *S. japonicum*. High IL-22 levels in cultures correlated with protection against hepatic fibrosis and portal hypertension. In our study, we focused on hepatic insulin sensitivity and observed expression of Th2-type and regulatory cytokines as well as target genes in the insulin pathway. High expression of IL-10, IL-13, IL-22, IL-33 and TGF-β were concomitant with high levels of IRS-1, IRS-2, INSR and GLUT4 in the liver after eggs were deposited in the tissue. We performed multiple linear regression analysis on these cytokines and their target genes in the insulin and glucose pathways through the course of schistosome infection and PZQ chemotherapy. Our data suggested that IL-22 and IL-33 were positively associated with enhanced insulin sensitivity and accelerated glucose metabolism in livers with chronic *S. japonicum* infection. After PZQ treatment, IL-13 and IL-22 had a similar effect on hepatic energy metabolism. Th2 and regulatory T-cell responses have been associated with protection against insulin resistance [20–22]. IL-13 plays a key role in regulating glucose homeostasis by modulating gluconeogenesis and may be a useful therapeutic target for treatment of diabetes and metabolic syndrome [31, 32]. IL-22 has a protective role in HFD-induced hepatic steatosis by regulating lipid metabolism in the liver [33]. Our study also showed that IL-13 and IL-22 can stimulate hepatic cell lines to express IRS-1, IRS-2, INSR, and GLUT4. These results suggest that anti-inflammatory cytokines are crucial in promoting the insulin-signalling cascade.

In addition to the effect of immune deviation and regulation induced by schistosomes and their derived molecules on host metabolism, we examined whether low energy metabolism can be memorized in patients with a schistosome infection, even in those receiving multiple PZQ treatments in endemic areas, and whether immune memory can be formed in patients with a previous schistosome infection and its effect on host metabolism. To date, the underlying molecular mechanisms of the interaction between immune memory and energy metabolism remain unknown. This issue is of practical significance and is worth further study.

## Conclusion

Our study demonstrated that mice who were infected with *Schistosoma japonicum* and subsequently received PZQ treatment had a significant improvement in their physiological status, maintained Th2 immune-deviation and had enhanced hepatic insulin sensitivity. This study will provide further insight into the relationship between schistosome infections and host metabolism.

## Additional files

Additional file 1: Figure S1. Dynamic liver pathology (HE, 100×) and a single egg-granulomatous area calculated at 12 weeks after PZQ or CMC treatment in Infection-Chemotherapy model mice. **a** C57BL/6-inf-CMC at 3 weeks post-treatment. **b** C57BL/6-inf-PZQ at 3 weeks post-treatment. **c** C57BL/6-inf-CMC at 6 weeks post-treatment. **d** C57BL/6-inf-PZQ at 6 weeks post-treatment. **e** C57BL/6-inf-CMC at 9 weeks post-treatment. **f** C57BL/6-inf-PZQ at 9 weeks post-treatment. **g** C57BL/6-inf-CMC at 12 weeks post-treatment. **h** C57BL/6-inf-PZQ at 12 weeks post-treatment. **i** C57BL/6-con group. **j** A single egg-granulomatous area calculated at 12 weeks after PZQ or CMC treatment. **P < 0.01 (TIFF 53679 kb)

Additional file 2: Figure S2. Serum AST, ALT, TG, TC, HDL-C and LDL-C concentrations at different time points after PZQ or CMC treatment in Infection-Chemotherapy model mice. * C57BL/6-inf-CMC group; # C57BL/6-inf-PZQ group *vs* C57BL/6-con group; C57BL/6-inf-PZQ group *vs* C57BL/6-inf-CMC group. *, # and & *P* < 0.05; **, ## and && *P* < 0.01 (TIFF 5287 kb)

Additional file 3: Table S1. Multivariable logistic regression analysis of hepatic inflammation for insulin signaling between groups of chronic infection and normal mice in Infection-Chemotherapy Model. **Table S2.** Multivariable logistic regression analysis of hepatic inflammation for insulin signaling between groups of PZQ chemotherapy and normal mice in Infection-Chemotherapy Model. **Table S3.** Multivariable logistic regression analysis of hepatic inflammation for insulin signaling between groups of chronic infection and PZQ chemotherapy in Infection-Chemotherapy Model. (DOCX 59 kb)

## Abbreviations

ALT: Alanine aminotransferase
AST: Aspartate aminotransferase
CMC: Carboxymethyl cellulose
CURES: The Chennai Urban Rural Epidemiology Study
ELISA: Enzyme-linked immunosorbent assay
G6PC: Glucose-6-phosphatase
GLUT4: Glucose transporter 4
GTT: Glucose-tolerance tests
HDL-C: High-density lipoprotein cholesterol
INSR: Insulin receptor
IRS-1: Insulin receptor substrate 1
IRS-2: Insulin receptor substrate 2
ITT: Insulin-tolerance tests
LDL-C: Low-density lipoprotein cholesterol
MetS: Metabolic syndrome
PSI: Previous schistosome infection
PZQ: Praziquantel
SEA: Soluble egg antigens
T2DM: Type 2 diabetes mellitus
TC: Total cholesterol
TG: Triglyceride
