# Supplementary figures and images for: Praziquantel treatment after Schistosoma japonicum infection maintains hepatic insulin sensitivity and improves glucose metabolism in mice
Source: Parasit Vectors. 2017 Oct 2;10:453. doi: 10.1186/s13071-017-2400-5 (PMC5625765; doi:10.1186/s13071-017-2400-5)

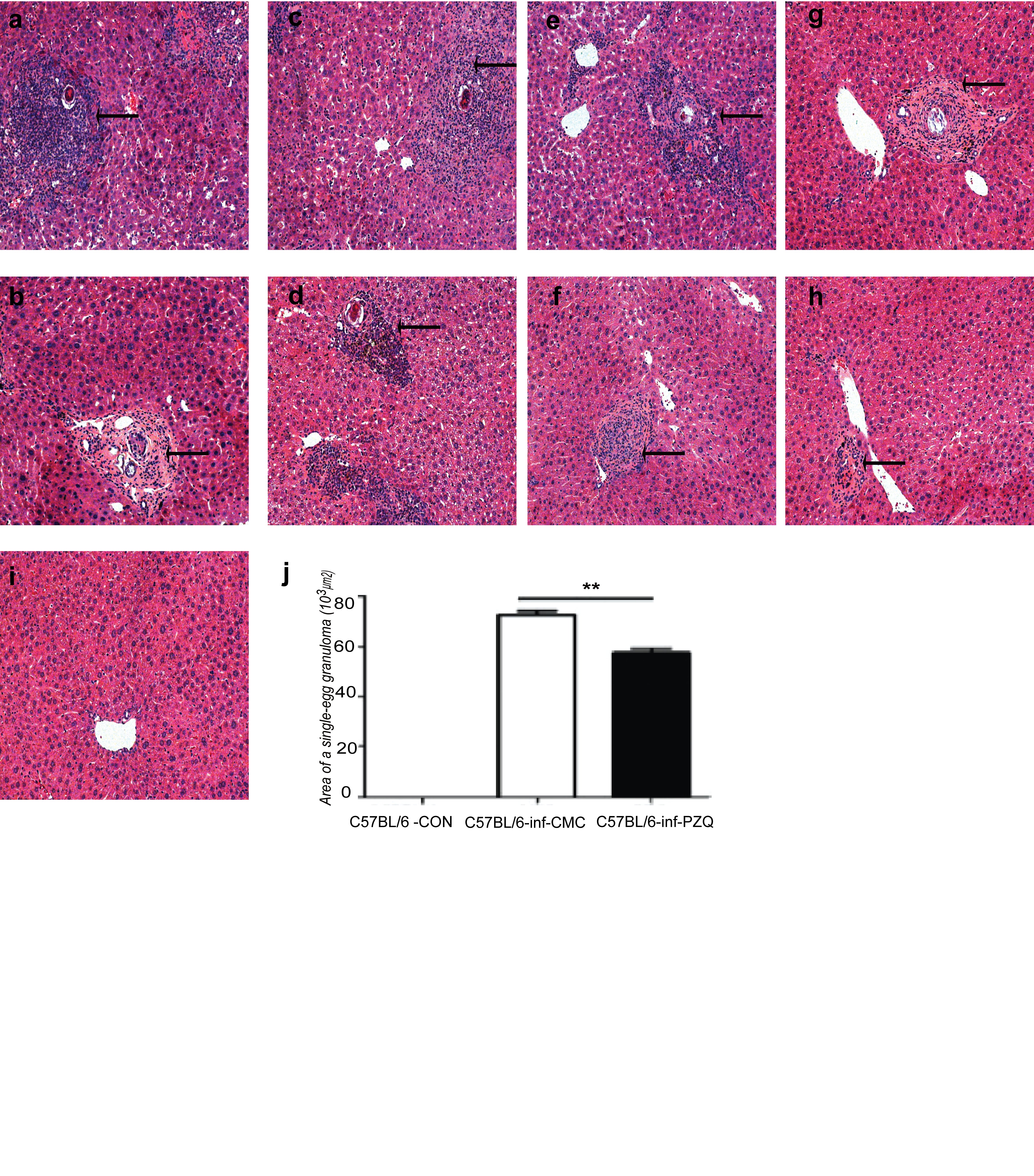

Supplement: Supplementary file 1 — Dynamic liver pathology (HE, 100×) and a single egg-granulomatous area calculated at 12 weeks after PZQ or CMC treatment in Infection-Chemotherapy model mice. a C57BL/6-inf-CMC at 3 weeks post-treatment. b C57BL/6-inf-PZQ at 3 weeks post-treatment. c C57BL/6-inf-CMC at 6 weeks post-treatment. d C57BL/6-inf-PZQ at 6 weeks post-treatment. e C57BL/6-inf-CMC at 9 weeks post-treatment. f C57BL/6-inf-PZQ at 9 weeks post-treatment. g C57BL/6-inf-CMC at 12 weeks post-treatment. h C57BL/6-inf-PZQ at 12 weeks post-treatment. i C57BL/6-con group. j A single egg-granulomatous area calculated at 12 weeks after PZQ or CMC treatment. **P < 0.01 (TIFF 53679 kb) [file 13071_2017_2400_MOESM1_ESM.tif]

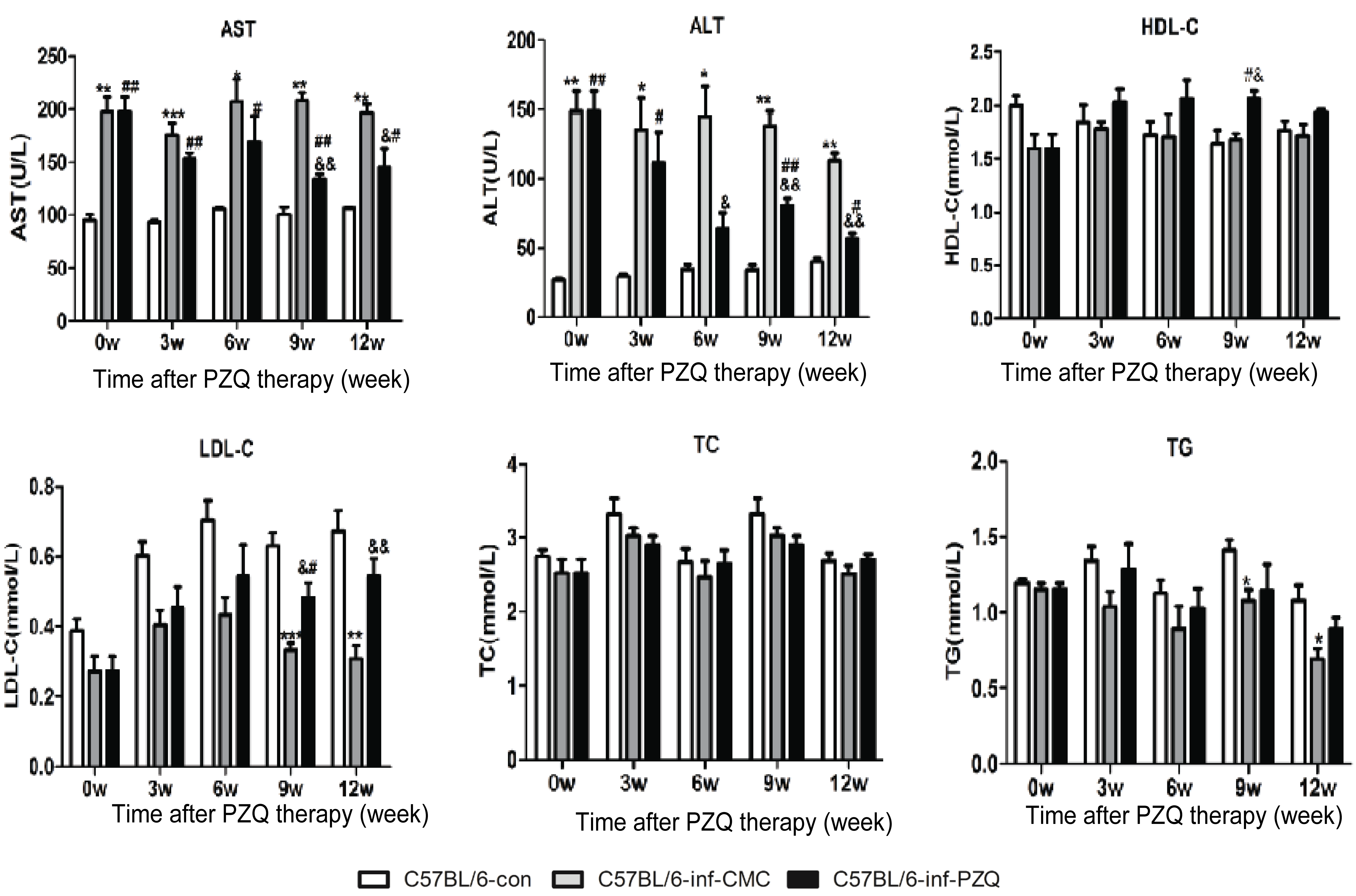

Supplement: Supplementary file 2 — Serum AST, ALT, TG, TC, HDL-C and LDL-C concentrations at different time points after PZQ or CMC treatment in Infection-Chemotherapy model mice. * C57BL/6-inf-CMC group; # C57BL/6-inf-PZQ group vs C57BL/6-con group; C57BL/6-inf-PZQ group vs C57BL/6-inf-CMC group. *, # and & P < 0.05; **, ## and && P < 0.01 (TIFF 5287 kb) [file 13071_2017_2400_MOESM2_ESM.tif]
