# Supplementary material for: Praziquantel treatment after Schistosoma japonicum infection maintains hepatic insulin sensitivity and improves glucose metabolism in mice
Source: Parasit Vectors. 2017 Oct 2;10:453. doi: 10.1186/s13071-017-2400-5 (PMC5625765; doi:10.1186/s13071-017-2400-5)
Supplement: Supplementary file 3 — Multivariable logistic regression analysis of hepatic inflammation for insulin signaling between groups of chronic infection and normal mice in Infection-Chemotherapy Model. Table S2. Multivariable logistic regression analysis of hepatic inflammation for insulin signaling between groups of PZQ chemotherapy and normal mice in Infection-Chemotherapy Model. Table S3. Multivariable logistic regression analysis of hepatic inflammation for insulin signaling between groups of chronic infection and PZQ chemotherapy in Infection-Chemotherapy Model. (DOCX 59 kb) [file 13071_2017_2400_MOESM3_ESM.docx]

**Table S1.** Multivariable logistic regression analysis of hepatic inflammation for insulin signaling between groups of chronic infection and normal mice in Infection-Chemotherapy Model

| Variables | IRS-1 | | | IRS-2 | | | INSR | | | G6PC | | | GLUT4 | | |
| --- | --- | --- | --- | --- | --- | --- | --- | --- | --- | --- | --- | --- | --- | --- | --- |
|  | *P* | B | 95%CI | *P* | B | 95%CI | *P* | B | 95%CI | *P* | B | 95%CI | *P* | B | 95%CI |
| TNF-α | 0.952 | 0.183 | -11.500-11.86 | 0.219 | 0.045 | -0.034-0.125 | 0.002 | 0.229 | 0.113-0.345 | 0.298 | -0.134 | -0.432-0.163 | 0.004 | -0.257 | -0.326--0.188 |
| IL-6 | 0.333 | -0.267 | -0.888-0.353 | 0.156 | -0.090 | -0.234-0.054 | 0.672 | -0.039 | -0.279-0.200 | 0.519 | -0.053 | -0.261-0.155 | 0.014 | -0.269 | -0.407--0.131 |
| IL-1β | 0.019 | 0.706 | 0.148-1.263 | 0.001 | -0.218 | -0.323- -0.112 | 0.002 | -0.325 | -0.490- -0.159 | 0.348 | -0.065 | -0.219-0.088 | 0.046 | 0.234 | 0.011-0.457 |
| IL-10 | 0.334 | 0.638 | -0.897-2.173 | 0.951 | -0.014 | -0.709-0.680 | 0.009 | -0.397 | -0.667- -0.128 | 0.014 | 0.316 | 0.082-0.550 | 0.028 | 0.560 | 0.150-0.970 |
| IL-13 | 0.824 | 0.213 | -2.269-2.694 | 0.603 | 0.012 | -0.041-0.064 | 0.665 | 0.008 | -0.034-0.050 | 0.190 | -0.046 | -0.122-0.030 | 0.737 | -0.022 | -0.656-0.612 |
| IL-22 | 0.000 | 0.947 | 0.834-1.061 | 0.311 | -0.039 | -0.129-0.050 | 0.015 | 0.118 | 0.030-0.206 | 0.020 | -0.112 | -0.202- -0.023 | 0.007 | 0.410 | 0.260-0.560 |
| IL-33 | 0.920 | -0.098 | -2.961-2.764 | 0.000 | 0.251 | 0.180-0.321 | 0.291 | 0.093 | -0.100-0.286 | 0.002 | -0.309 | -0.477- -0.142 | 0.018 | 0.285 | 0.120-0.449 |
| TGF-β | 0.144 | -0.705 | -1.720-0.310 | 0.184 | -0.095 | -0.246-0.055 | 0.177 | -0.967 | -2.548-0.614 | 0.361 | 0.088 | -0.121-0.297 | 0.012 | -0.850 | -1.253- -0.448 |

**Table S2.** Multivariable logistic regression analysis of hepatic inflammation for insulin signaling between groups of PZQ chemotherapy and normal mice in Infection-Chemotherapy Model

| Variables | IRS-1 | | | IRS-2 | | | INSR | | | G6PC | | | GLUT4 | | |
| --- | --- | --- | --- | --- | --- | --- | --- | --- | --- | --- | --- | --- | --- | --- | --- |
|  | *P* | B | 95%CI | *P* | B | 95%CI | *P* | B | 95%CI | *P* | B | 95%CI | *P* | B | 95%CI |
| TNF-α | 0.860 | -0.004 | -0.490- -0.420 | 0.938 | -0.001 | -0.024-0.023 | 0.540 | -0.006 | -0.025-0.014 | 0.018 | -0.019 | -0.034- -0.004 | 0.995 | 0.232 | -0.048-0.048 |
| IL-6 | 0.870 | -0.170 | -2.452-2.114 | 0.001 | -1.085 | -1.645- -0.526 | 0.846 | -0.059 | -0.719-0.601 | 0.545 | -0.127 | -0.580-0.325 | 0.360 | 0.584 | -0.771-1.939 |
| IL-1β | 0.252 | 0.432 | 0.346-1.251 | 0.023 | 0.170 | 0.028-0.311 | 0.032 | -0.250 | -0.475- -0.025 | 0.028 | 0.120 | 0.015-0.225 | 0.035 | 0.597 | 0.050-1.145 |
| IL-10 | 0.000 | -1.990 | -2.790 - -1.189 | 0.000 | -0.845 | -1.219- -0.470 | 0.246 | -0.067 | -0.187-0.052 | 0.366 | 0.107 | -0.143-0.357 | 0.002 | -1.041 | -1.623- -0.460 |
| IL-13 | 0.000 | 0.262 | 0.157-0.367 | 0.003 | 0.078 | 0.033-0.122 | 0.816 | 0.008 | -0.064-0.080 | 0.721 | -0.003 | -0.017-0.012 | 0.004 | 0.140 | 0.055-0.225 |
| IL-22 | 0.000 | 0.305 | -0.241-0.369 | 0.882 | -0.004 | -0.057-0.050 | 0.005 | 0.055 | 0.020-0.090 | 0.278 | -0.014 | -0.042-0.013 | 0.044 | 0.089 | 0.003-0.176 |
| IL-33 | 0.777 | 0.092 | -0.614-0.789 | 0.557 | 0.037 | -0.098-0.170 | 0.129 | 0.080 | -0.026-0.185 | 0.066 | 0.114 | -0.009-0.237 | 0.000 | 0.547 | 0.306-0.788 |
| TGF-β | 0.447 | 0.468 | -0.898-1.899 | 0.000 | 1.352 | 0.794-1.910 | 0.598 | 0.106 | -0.585-0.958 | 0.958 | -0.015 | -0.624-0.594 | 0.514 | -0.335 | -1.430-0.760 |

**Table S3.** Multivariable logistic regression analysis of hepatic inflammation for insulin signaling between groups of chronic infection and PZQ chemotherapy in Infection-Chemotherapy Model

| Variables | IRS-1 | | | IRS-2 | | | INSR | | | G6PC | | | GLUT4 | | |
| --- | --- | --- | --- | --- | --- | --- | --- | --- | --- | --- | --- | --- | --- | --- | --- |
|  | *P* | B | 95%CI | *P* | B | 95%CI | *P* | B | 95%CI | *P* | B | 95%CI | *P* | B | 95%CI |
| TNF-α | 0.544 | -0.059 | -0.265- -0.146 | 0.257 | 0.038 | -0.031-0.108 | 0.013 | -0.339 | -0.595- -0.084 | 0.000 | 0.418 | 0.271-0.565 | 0.000 | 0.806 | 0.528-1.084 |
| IL-6 | 0.776 | 0.457 | -2.994-3.909 | 0.763 | -0.086 | -0.685-0.513 | 0.002 | 1.287 | 0.574-2.001 | 0.000 | -2.014 | -2.424- -1.604 | 0.000 | -2.378 | -3.480- -1.275 |
| IL-1β | 0.001 | 0.970 | 0.468-1.471 | 0.543 | 0.342 | -0.857-1.540 | 0.745 | -0.142 | -1.070-0.786 | 0.000 | 2.048 | 1.661-2.434 | 0.000 | 1.886 | 1.246-2.526 |
| IL-10 | 0.279 | -0.262 | -0.759-0.235 | 0.000 | 0.494 | 0.335-0.653 | 0.035 | -0.617 | -1.184- -0.049 | 0.539 | -0.197 | -0.879-0.486 | 0.001 | 1.468 | 0.712-2.225 |
| IL-13 | 0.000 | 0.274 | 0.176-0.372 | 0.670 | -0.026 | -0.158-0.105 | 0.002 | 0.248 | 0.110-0.385 | 0.023 | -0.091 | -0.168- -0.014 | 0.112 | 0.114 | -0.031-0.260 |
| IL-22 | 0.141 | -0.082 | -0.194-0.030 | 0.004 | 0.083 | 0.031-0.135 | 0.507 | 0.053 | -0.117-0.222 | 0.172 | -0.080 | -0.199-0.040 | 0.642 | 0.030 | -0.109-0.169 |
| IL-33 | 0.381 | -0.562 | -1.895-0.771 | 0.365 | 0.103 | -0.131-0.338 | 0.003 | -1.633 | -2.581- -0.684 | 0.000 | 1.311 | 0.882-1.740 | 0.001 | 1.921 | 0.949-2.892 |
| TGF-β | 0.852 | 0.061 | -0.636-0.758 | 0.746 | -0.058 | -0.436-0.321 | 0.002 | 0.770 | 0.329-1.211 | 0.405 | -0.159 | -0.557-0.240 | 0.000 | -1.078 | -1.481- -0.675 |
